# Innate Immune Defenses in Human Tuberculosis: An Overview of the Interactions between *Mycobacterium tuberculosis* and Innate Immune Cells

**DOI:** 10.1155/2015/747543

**Published:** 2015-07-14

**Authors:** Jonathan Kevin Sia, Maria Georgieva, Jyothi Rengarajan

**Affiliations:** ^1^Emory Vaccine Center, Emory University, Atlanta, GA 30329, USA; ^2^Division of Infectious Diseases, Department of Medicine, Emory University School of Medicine, Emory University, Atlanta, GA 30329, USA

## Abstract

Tuberculosis (TB) remains a serious global public health problem that results in up to 2 million deaths each year. TB is caused by the human pathogen, *Mycobacterium tuberculosis* (Mtb), which infects primarily innate immune cells patrolling the lung. Innate immune cells serve as barometers of the immune response against Mtb infection by determining the inflammatory milieu in the lungs and promoting the generation of adaptive immune responses. However, innate immune cells are also potential niches for bacterial replication and are readily manipulated by Mtb. Our understanding of the early interactions between Mtb and innate immune cells is limited, especially in the context of human infection. This review will focus on Mtb interactions with human macrophages, dendritic cells, neutrophils, and NK cells and detail evidence that Mtb modulation of these cells negatively impacts Mtb-specific immune responses. Furthermore, this review will emphasize important innate immune pathways uncovered through human immunogenetic studies. Insights into the human innate immune response to Mtb infection are necessary for providing a rational basis for the augmentation of immune responses against Mtb infection, especially with respect to the generation of effective anti-TB immunotherapeutics and vaccines.

## 1. Introduction

Infection with* Mycobacterium tuberculosis* (Mtb), the causative agent of tuberculosis (TB), was responsible for 1.5 million deaths and 9 million cases of TB in 2013, according to the World Health Organization [1]. While only 5–10% of individuals infected with Mtb progress to active TB disease, approximately one-third of the world population, or over 2 billion people, are estimated to have latent Mtb infection (LTBI) [2]. Latently infected individuals control Mtb infection and are clinically asymptomatic but retain a significant risk of progressing to TB by reactivation of latent Mtb when immune compromised [3]. This is due to the ability of Mtb to persist within granulomatous lesions in the lungs of individuals and the inability of host immunity to completely eradicate mycobacteria from host tissues [4]. Granuloma formation is initiated by Mtb-infected macrophages and continues with the development of multinucleated giant cells (MGCs) and lipid-filled foamy macrophages surrounded by a ring of lymphocytes encapsulated in a fibrotic cuff [5, 6]. Although macrophages and T cells play a central role in the formation of the granuloma, the complete cellular composition of the human granuloma throughout Mtb infection remains unclarified and other cell types, including dendritic cells (DCs), neutrophils, and B lymphocytes, are present and have been shown to contribute to cellular recruitment and the maturation of the granuloma [7]. Thus, granulomas are a testament to the involvement of both innate and adaptive immune cells in the human immune response to TB. Huge strides have been made towards understanding the acute and chronic T cell response to Mtb infection from studies in animal models, but the earliest encounters between Mtb and the human innate immune system are incompletely understood.

Investigations in animal models of TB, human clinical and epidemiological studies on the genetics of mycobacterial susceptibility, and* in vitro* work with primary human cells strongly indicate that innate immune responses play a major role in determining the outcome of TB by helping control bacterial load and through shaping the nature and magnitude of adaptive immune responses [8–10]. While innate antimicrobial pathways that are activated early and throughout infection play a role in limiting disease, myeloid cells also serve as the primary niches for Mtb replication. Moreover, Mtb has evolved multiple strategies to modulate innate immune responses and prevent optimal activation of adaptive immunity. Thus, understanding the crosstalk between innate and adaptive immune cells in human TB is critical for identifying novel targets of immunomodulatory therapies and for elucidating mechanisms of protective immunity. However, innate immune responses to Mtb infection in humans remain relatively poorly understood, largely because of the inherent difficulties in studying lung-specific immunity in humans. This review will focus on the key innate immune cell types implicated in the human response to Mtb, the interaction of innate immune cells with Mtb, and their influence on adaptive immune responses and the course of disease. We will also review specific human immunogenetics studies that link perturbations in innate immunity to mycobacterial susceptibility.

## 2. Friendly Guardians: Innate Immune Cells in TB

The major innate cell types that have been studied in humans are macrophages, neutrophils, DCs, and natural killer (NK) cells. Recently, other cell types not classically defined as immune cells, such as airway epithelial cells, have been shown to contribute to the immune response against Mtb in animal models of TB [11] and* in vitro* studies with human cell lines [12]. However, this review will focus on the roles of the classically defined innate immune cells in human TB as these cells serve as both the primary cellular niches for Mtb replication as well as the initial sources of immune pressure to contain infection.

### 2.1. Macrophages

**Figure 1:**
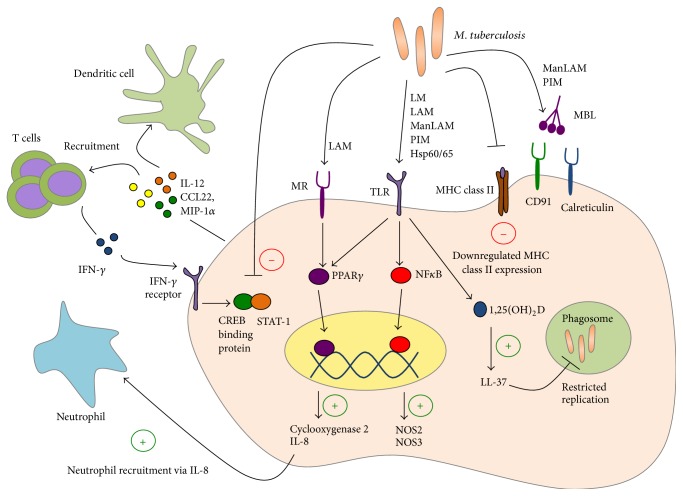
Human alveolar macrophages possess an array of receptors to recognize* M. tuberculosis* (Mtb). In response to Mtb infection, macrophages upregulate effector and signalling pathways to both prevent bacterial replication and recruit other immune cells into the site of infection.* M. tuberculosis* components, including lipoarabinomannans (LAMs), lipomannans (LMs), phosphatidylinositol mannosides (PIMs), and heat shock proteins (HSPs), are recognized by a variety of pattern recognition receptors. Following recognition of Mtb, host effectors, such as NF-*κ*B and PPAR*γ*, are activated to upregulate antimicrobial factors. These antimicrobial peptides (e.g., LL-37) possess both effector and signalling functions to actively interfere with bacterial replication as well as recruit and activate neutrophils, dendritic cells, and T cells. However, Mtb interferes with macrophage effector and signalling pathways. Most importantly, Mtb downregulates MHCII expression on macrophages to prevent optimal interaction with antigen specific T-cells. Furthermore, Mtb interferes with IFN*γ* signaling, a T cell cytokine mediator critical for upregulating the inherent antimicrobial capacity of macrophages during infection.

Alveolar macrophages are one of the first host cell types to encounter Mtb in the lungs following aerosol transmission. While macrophages function as the first line of defense against Mtb infection, early interactions between macrophages and Mtb favor the bacteria. Thus, macrophages are a major cellular niche for bacterial replication during early infection and serve as reservoirs for persistent bacteria within the lung granulomas during chronic infection. In human TB, several aspects of macrophage functions have been investigated, including phagocytosis of bacteria, induction of antimicrobial pathways, and responsiveness to interferon gamma (IFN-*γ*) (Figure 1).

A number of receptors recognizing a wide variety of mycobacterial ligands play a role in human macrophage phagocytosis of Mtb. Collectins (e.g., surfactant proteins A and D and mannose-binding lectin), C-type lectins (e.g., mannose receptor, DC-SIGN, and Dectin-1), toll-like receptors (TLRs; e.g., TLR-2, TLR-4, and TLR-9), and many others have been implicated in the recognition and uptake of mycobacterial glycolipids, lipoproteins, and carbohydrates [10]. Of these, the best characterized are mannose-binding lectin (MBL) and mannose receptor (MR). MBL belongs to a family of soluble C-type lectins, called collectins, which are involved in the recognition and clearance of apoptotic cells via calreticulin and CD91 mediated phagocytosis [13]. During Mtb infection, MBL recognizes mannosylated lipoarabinomannan (ManLAM) and phosphatidylinositol mannosides (PIMs) [10]. In addition to recognition of Mtb ligands and phagocytosis, macrophage receptors are also involved in the activation of specific downstream pathways. As an example, MR, a transmembrane C-type lectin, ligates Mtb lipoarabinomannan and activates macrophage peroxisome proliferator activated receptor gamma (PPAR*γ*) expression in a phospholipase A2 and TLR-2 dependent manner [14, 15]. In contrast to the avirulent vaccine strain* Mycobacterium bovis* Bacillus Calmette-Guérin (BCG), virulent Mtb activates macrophage PPAR*γ* and induces the production of cyclooxygenase 2 and IL-8, which regulate inflammatory responses via arachidonic acid metabolites and the recruitment of neutrophils, respectively [14]. These studies suggest that the earliest Mtb interactions with macrophages at the level of receptor-mediated phagocytosis can influence the ensuing inflammatory response. Moreover, it is likely that Mtb manipulates these responses in order to promote its survival and dissemination. No single receptor has been demonstrated to be essential for macrophage phagocytosis of Mtb during human infection and it is clear that Mtb is recognized by numerous receptors which induces a network of coordinated receptor-mediated signaling pathways that lead to distinct gene expression profiles of infected macrophages at different stages of disease. Studies centered on the gene expression profiles of Mtb-infected macrophages have largely been explored in murine cells, though a few studies have examined proinflammatory cytokine profiles in Mtb-infected human macrophages [16, 17] as well as global gene expression after infection of* in vitro* blood monocyte-derived macrophages or human monocytic cell lines with Mtb [18–22]. These early gene profiling studies in infected human macrophages provided evidence for the importance of IFN-*γ* transcription in suppressing Mtb gene expression [17], highlighted a prominent role for IL-1*β* and other proinflammatory cytokines at early and late timepoints after infection, and showed that macrophage responses to pathogenic mycobacteria differed from responses to infection with nonpathogenic mycobacteria [18–22]. Additionally, transcriptional profiling studies of blood monocyte-derived human macrophages after Mtb infection* in vitro* have provided corollary evidence for the importance of known factors such as IL-12 in combating Mtb as well as new insights into other factors not known to be important previously, including macrophage-derived chemokine CCL22 (MDC) and macrophage inflammatory protein-1*α* (MIP-1*α*/CCL3) [17]. Importantly, a study that examined gene expression of* ex vivo* stimulated macrophages from TB patients resulted in the association of CCL1 with TB susceptibility [23]. Studies that investigate gene expression profiles of primary macrophage cells from patients with TB are limited but are required to yield the most relevant insights into how Mtb interacts with human macrophages* in vivo*. With a few exceptions, the gene expression studies in Mtb-infected human macrophages and macrophage cell lines highlighted here have validated and supplemented data derived from other mechanistic studies in human monocytic cell lines and mouse cells. Much remains unknown about the gene expression profile of Mtb-infected primary macrophages from TB patients and studies utilizing primary samples from TB cohorts will provide the best insight into the human macrophage response to Mtb infection* in vivo*.

Insights into the survival and replication of Mtb within macrophage phagosomes have largely been derived from studying murine macrophages, and studies on Mtb manipulation of phagosomal function in human macrophages have been relatively limited. Interestingly, recent studies have examined alveolar macrophages from the bronchoalveolar lavage (BAL) of patients coinfected with Mtb and HIV [24, 25] and demonstrate that Mtb resides within relatively nonacidified compartments in otherwise functionally capable macrophages. This suggests that the phagosome in human macrophages is specifically modulated by Mtb to make it a preferential niche and further studies will be needed to clarify mechanisms of immune evasion that specifically target the human macrophage phagosome. Tools developed for the assessment of innate immune functions, including vacuole acidification and superoxide burst, will be important in answering questions as to why bacteria are able to replicate within otherwise hostile environments [26, 27]. The IFN-*γ* pathway remains a critical pathway for resistance against mycobacterial infection, as highlighted by increased susceptibility to mycobacterial infections in humans with genetic impairments in the IL-12/STAT-1 pathway [8, 9, 28, 29], though* in vitro* evidence indicates that IFN-*γ* alone does not fully limit Mtb replication in human macrophages and that Vitamin D signaling pathways augment macrophage IFN-*γ* responsiveness [30–37]. Vitamin D was shown to act synergistically with IFN-*γ* to augment antimycobacterial activity in human monocytes [37]. Vitamin D treatment enhances a variety of important downstream pathways in macrophages, including autophagy, phagosomal maturation, and the production of antimicrobial peptides [30, 35]. Additional studies have demonstrated that the bioactive 1,25-dihydroxyvitamin D3 can restrict Mtb replication within infected human macrophages [36] in a phosphatidylinositol 3-kinase-dependent [34] and TLR-dependent [33] manner. Mechanistically, Vitamin D upregulates gene expression of macrophage* hCAP-18*, which encodes for the antimicrobial peptide LL-37 (cathelicidin), and LL-37 trafficking to Mtb-containing phagosomes is purported to mediate the antimycobacterial effects of Vitamin D [31, 32].

Mtb contains numerous pathogen associated molecular patterns (PAMPs) that are recognized by a variety of cell surface and intracellular pattern recognition receptors (PRRs) on macrophages. Engagement of PRRs leads to activation of antimicrobial effector functions within the macrophage. For example, TLR2 recognizes mycobacterial mannosylated lipoarabinomannans and engagement of this receptor-ligand pair leads to downstream NF-*κ*B activation and inducible nitric oxide synthase (*iNOS*) gene transcription [38]. NOS2 and NOS3 expression has been implicated in the production of nitric oxide (NO) in human macrophages [39] and clear induction of macrophage NOS2 mRNA can be seen in the BAL of TB patients compared to healthy controls [35, 40]. In contrast to murine studies, NO seems to have limited bactericidal or bacteriostatic effects against Mtb during* in vitro* infection of human alveolar macrophages and primary monocytes post-IFN-*γ* treatment [39, 41], suggesting that the critical immune responses to Mtb garnered from studies in animal models may not be as important during human infection. Alternatively, antimycobacterial effects of NO may in fact occur* in vivo* within the lung microenvironment.

The IFN-*γ*/IL-12 axis is critical in host resistance to Mtb in mice and in humans [42–46]. Clinical observations of increased levels of IFN-*γ* in the pleural fluid and BAL of patients with confirmed pulmonary TB compared to healthy controls suggest that IFN-*γ* plays a prominent role in human TB infection [47–50]. While murine macrophages activated by IFN-*γ* alone show distinctly augmented capacity for antimycobacterial functions compared to untreated macrophages, human macrophages require additional factors such as Vitamin D, in addition to IFN-*γ*, to maximize antimycobacterial functions [37]. This is perhaps due to Mtb-mediated inhibition of critical STAT1 protein-protein interactions with cAMP response element binding (CREB) binding protein [51–53], leading to hyporesponsiveness to IFN-*γ* stimulation. Additionally, MHC class II expression, normally upregulated after IFN-*γ* activation of macrophages, is downregulated after Mtb infection of human macrophages via decreased expression of class II transactivator (CIITA) [52, 54, 55]. This may play a role in dampening adaptive immune responses by attenuating T cell recognition of infected macrophages and could explain the reported defects in antigen recognition by Mtb-specific lymphocytes in the granuloma [56].

Overall, macrophages are clearly at least capable of restricting Mtb bacilli given appropriate activation signals from antigen specific T cells and the local lung microenvironment. However, questions remain regarding whether effective juxtaposition of infected macrophages and activated T cells occurs within the confines of the lung. Studies aimed at answering basic questions about infected macrophages in human TB, including signaling pathways subverted during infection, the activation status of Mtb-infected human alveolar macrophages, and the crosstalk between macrophages and T cells within infected lungs, are critical for developing immunomodulatory therapies for TB.

### 2.2. Neutrophils

**Figure 2:**
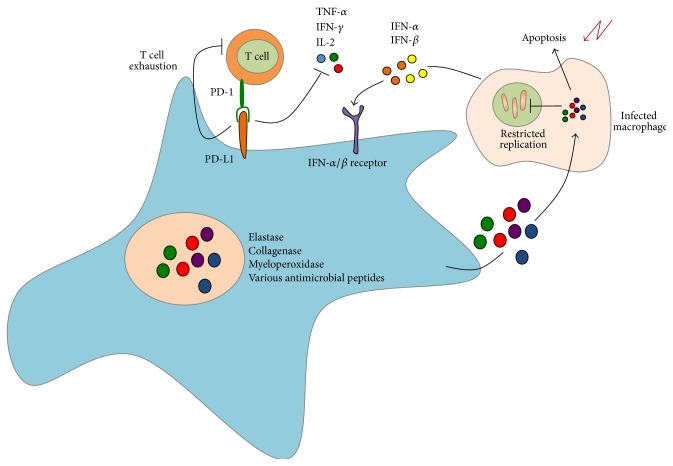
Neutrophils constitute a major subset of innate immune cells in the BAL and sputum of patients with active pulmonary TB. During infection with* M. tuberculosis*, neutrophils produce and secrete a variety of antimicrobial enzymes to restrict bacterial growth within infected macrophages. These neutrophil effectors promote apoptosis of infected macrophages, thereby limiting Mtb survival within infected host cells. However, these enzymes also mediate lung tissue damage and sustained, hyperactivated inflammatory response. Furthermore, transcriptional profiling studies have demonstrated the importance of PD-L1, a cell-surface associated molecule, in modulating T cell responses during infection with Mtb. Additional transcriptional studies have identified a blood based IFN-inducible gene signature in neutrophils that is unique to tuberculosis-specific immune responses.

During infection, neutrophils phagocytose bacteria discharge antimicrobial effectors from their granules and constitute a potent population of effector cells that can mediate both antimycobacterial activity and immunopathology in human TB (Figure 2). This is because release of factors such as elastase, collagenase, and myeloperoxidase by neutrophils during their respiratory burst indiscriminately damages bacterial and host cells alike. Neutrophils are the most abundant cell type found in the BAL and sputum of active pulmonary TB patients and are second only to lymphocytes within lungs [57]. One study found an inverse correlation between the development of pulmonary TB and the number of neutrophils in peripheral blood of contacts of active TB patients and* in vitro* depletion of neutrophils from whole blood led to poor induction of antimicrobial peptides (AMPs) and failure to restrict BCG and Mtb growth [58]. Apoptotic neutrophils and purified neutrophil granules, both of which still contain active antimicrobial peptides, have been demonstrated to be taken up by infected macrophages and can lead to impairment of bacterial replication* in vitro* [59].

Apart from their degranulation capacity, neutrophils have recently been implicated in a more immunoregulatory role during Mtb infection. Interaction between programmed death ligand 1 (PD-L1) on myeloid cells and programmed death receptor (PD-1) on lymphocytes is thought to promote the development of dysfunctional, or exhausted, lymphocyte responses during chronic infections [60–62]. Recent transcriptional profiling studies determined that cell surface expression of programmed death ligand 1 (PD-L1) by neutrophils was primarily responsible for high levels of PD-L1 expression in whole blood of active TB patients [63]. Another study described a 393 blood-based transcript signature that differentiated active TB infection from healthy individuals with LTBI. From this, the authors derived an 86-gene signature that corresponded to neutrophil expression of type I and type II interferon inducible genes that distinguished active TB infection from other inflammatory conditions [64]. It will be important to extend such global transcriptional analyses to the lung, which is the primary site of Mtb infection in pulmonary TB. Indeed, a recent study utilizing biopsy samples from a variety of human tissues to investigate the steady-state T cell compartment in different places throughout the body demonstrated that different tissue compartments, including the lung, contained distinct T cell populations [65] and it is likely that the same principles apply to innate immune populations during states of infection. Since neutrophils comprise a significant percentage of cells that infiltrate the lung during human TB, it will be important to determine their roles in lung tissue and their contribution to uncontrolled inflammation and immunopathology.

### 2.3. Dendritic Cells

**Figure 3:**
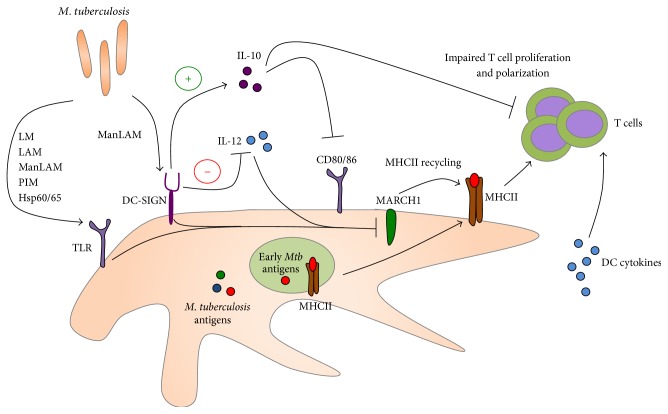
DCs are the primary antigen presenting cells (APCs) in the immune system and play a central role in activation and differentiation of T cells by presenting antigenic peptides. DCs recognize a variety of* M. tuberculosis* components directly primarily through TLRs and DC-SIGN. Mtb impacts DC maturation and CD80/CD86 expression via induction of the immunosuppressive IL-10 mediator. Furthermore, engagement of TLRs and DC-SIGN during Mtb infection downregulates MARCH1, a ubiquitin ligase critical for recycling of MHCII on the cell surface. Downregulation of MARCH1 early during infection may play a role in limiting the repertoire of presented Mtb antigens and narrows the adaptive immune response in human TB.

DCs are critical cell types involved in bridging innate and adaptive immunity. DCs are the primary antigen presenting cells that initiate adaptive immune responses through their capacity to present antigen, their costimulatory capacity, and secretion of T-helper polarizing cytokines (Figure 3). In mouse models of TB, it has been shown that DCs constitute a significant population of cells harboring Mtb* in vitro* and* in vivo* [66, 67]. However, whether or not human DCs serve as a major cellular niche for Mtb replication* in vivo* remains unclear.* In vitro* studies in monocyte-derived DCs suggest low levels of bacterial replication within these DCs [68], but further studies are needed to substantiate these observations. Monocyte-derived human DCs express mannose receptors, CD11b, CD11c, and DC-SIGN, all of which are capable of recognizing Mtb ligands. Indeed, DC-SIGN has been shown to serve as a major receptor for Mtb entry into DCs via recognition of ManLAM [69]. Under homeostatic conditions, DC-SIGN functions by binding ICAM-2 on endothelial surfaces to allow for efficient DC migration. During Mtb infection, ligation of DC-SIGN by Mtb ManLAM leads to the induction of the anti-inflammatory cytokine IL-10, which has been implicated in the impairment of DC maturation and expression of costimulatory molecules [70]. Other studies suggest that ManLAMs are capable of inducing a negative signal that inhibits IL-12 production through both mannose receptor and DC-SIGN [71]. These data suggest that Mtb may be modulating DC functions in order to prevent optimal induction of host adaptive immunity.

Subversion of DC functions by Mtb represents an ideal strategy for slow growing Mtb to evade adaptive immunity. Manipulation of DC maturation, cytokine production, and antigen presentation will affect the kinetics, nature, and magnitude of the T cell response and can provide Mtb with time to establish a foothold within the lungs. Studies that show impaired ability of Mtb-infected monocyte-derived DCs to stimulate lymphoproliferation of naïve and memory CD4s and CD8s provide strong evidence suggesting that Mtb infection of DCs can impair T cell responses during human infection [72]. Since Mtb is a slow growing organism, antigen availability, especially at the early stages of infection, may be an additional reason for poor T cell responses. Under homeostatic conditions, DCs are able to retain peptide-MHC complexes at the cell surface much more efficiently than macrophages, primarily due to the downregulation of membrane associated RING-CH-1 protein (MARCH1), a ubiquitin E3 ligase that helps recycle cell surface MHC complexes [73, 74]. MHC class II cycling from the phagosome to the plasma membrane is induced by DC maturation when TLRs first engage Mtb ligands but may occur before the availability of loadable Mtb antigens, thereby leading to Mtb immune evasion from CD4 T cells [75]. These data might suggest that Mtb antigens are not properly represented during the initiation of adaptive immune responses and may lead to an overabundance of antigen specific T cells that are specific for antigens that may not be relevant at different stages of infection. It has been previously shown that BCG vaccination fails to elicit human T cell responses to latency associated Mtb antigens [76] and vaccination strategies implementing latency associated antigens have shown some promise in the mouse model [77]. The DC plays a central role in the presentation of any Mtb antigen throughout infection and future studies must look to the DC in order to understand why certain antigens are under or nonrepresented at the T cell level. Many questions remain regarding the mechanisms that Mtb employs to manipulate DCs and the subsequent consequences of that manipulation on the nature, kinetics, and magnitude of the adaptive immune response. Studies in humans will remain limited as DCs are poorly represented in BAL, and peripheral blood derived DCs may not be representative of DCs found in the lungs. Nevertheless, it will be important to pursue mechanistic studies on lung DC biology during Mtb infection in humans as well as in animal models of TB, including in mice and nonhuman primates where immunological reagents are readily available.

### 2.4. Natural Killer Cells

**Figure 4:**
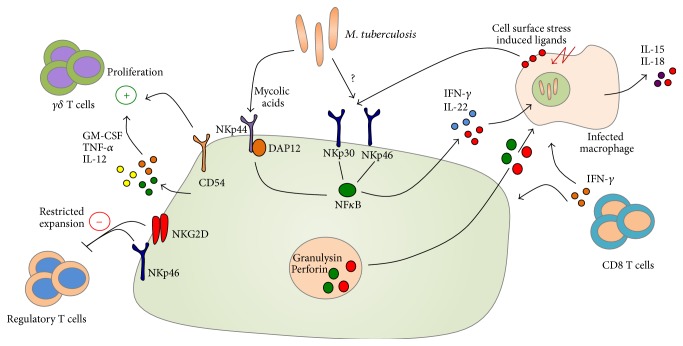
Natural killer (NK) cells have the capacity to restrict* M. tuberculosis* replication through the production of soluble mediators such as GM-CSF, IL-12, TNF-*α*, IL-22, and IFN-*γ*. These upregulate the antimicrobial function of infected macrophages and activate antigen-specific T cell responses during* M. tuberculosis* infection. NK cell-derived antimicrobial factors such as granulysin and perforin indirectly restrict Mtb growth via the lysis of infected host cells. Several studies suggest that NK cells directly recognize Mtb-derived mycolic acids via NKp44. Aside from direct recognition of Mtb ligands, NKp30 and NKp46 recognize a variety of stress molecules upregulated on the surface of infected host cells.

Natural killer (NK) cells are granular innate lymphocytes possessing potent cytolytic capacity. NK cells act early during infection, are not MHC-restricted, and depend upon licensing based on engagement of various activating receptors found on their cell surface by ligands upregulated by stressed or infected target cells (Figure 4). Various Mtb cell wall components, such as mycolic acids, are direct ligands for the natural cytotoxicity receptor (NCR) NKp44 on NK cells [78], and human NK cells exhibit the capacity to lyse Mtb-infected macrophages* in vitro* [79, 80]. Additionally, NK cells can also produce IFN-*γ* and IL-22, which can inhibit intracellular growth of Mtb* in vitro* by enhancing phagolysosomal fusion [81], or can promote the production of IFN-*γ* from CD8 T cells by stimulating IL-15 and IL-18 production from Mtb-infected monocytes* in vitro* [82].

Studies on the functionality of NK cells in human TB are limited, but there are indications that NK cells may be functionally impaired during TB. Patients newly diagnosed with pulmonary TB display decreased frequencies of NK cell subsets, coinciding with lowered expression of NKp30, NKp46, and IFN-*γ* [83]. Anti-TB treatment regimens leading to reductions in mycobacterial load have been shown to partially restore cytolytic capabilities of NK cells [84]. Furthermore, NK cells in patients with tuberculous pleurisy express high levels of ICAM-1 [85], important for the establishment of the immunological synapse, chemokine receptors, and TLR expression [86], and are able to activate autologous lymphocytes under* ex vivo* conditions [85].

In addition to direct killing, NK cells can also promote *γδ* T cell proliferation via CD54, TNF*α*, GM-CSF, and IL-12 [87] and, upon recognition of an NK ligand, ULBP1 can restrict the expansion of regulatory T cells in an NKG2D/NKp46 dependent manner [88]. These cells are sensitive to the local microenvironment and monocyte produced IL-10 has been shown to impair NK cell lytic capacity and decrease expression of activating NK cell receptors [89]. Very little is known about NK cells in human TB, but evidence suggests that they play a role in restricting bacterial growth indirectly, via promotion of CD8 [82] and *γδ* T cell responses [87], and directly, via killing of Mtb-infected monocytes and macrophages [79, 80].

The success of Mtb infection likely hinges upon its early interactions with cells of the innate immune system. Macrophages and neutrophils can take up and kill bacteria but can be subverted by Mtb to promote chronic inflammatory conditions harmful to the lung. Additionally, DCs are central to the generation of Mtb-specific T cells that can bolster immunity but are manipulated to establish poor or misdirected T cell responses. NK cells are capable of directly and indirectly promoting killing of Mtb, but their functional capacity is diminished and little is known about how well they are activated during infection. Each cell type has distinct roles to play in defending the host against Mtb, but they are also readily coopted into helping Mtb establish a long term infection. The difficulties of* in vivo* and lung* in situ* human studies are major roadblocks towards the understanding of innate immune responses during Mtb infection, but population based immunogenetics studies can offer important insights into innate immune pathways critical for antimycobacterial immunity.

## 3. Human Innate Immunogenetics and Mycobacterial Susceptibility

Historically, our knowledge regarding human innate and adaptive immune pathways involved during Mtb infection stems from clinical observations in patients suffering from Mendelian Susceptibility to Mycobacterial Diseases (MSMD) and then validated in murine models of TB. Patients suffering from MSMD have genetic polymorphisms that predispose them to infections with various environmental mycobacteria as well as infection with classically avirulent mycobacteria such as BCG, though a significant portion of MSMD patients also suffer from disseminated TB [44]. The importance of innate immunity in combating mycobacterial infections is highlighted in patients with mutations in two innate immune autosomal genes (*IL12B* and* IL12Rβ1*), who suffer widespread and recurrent mycobacterial infections early in life [8, 28, 29]. Fortunately, individuals with polymorphisms in the IL-12 locus can receive treatment with exogenous IL-12 and are less likely to suffer from fatal infections, highlighting a role for macrophage and/or DC-derived IL-12 in the generation of IFN-*γ* responses that control infection [9]. Mutations in* IL12Rβ1* are among the most common genetic factors associated with MSMD resulting in susceptibility to primary mycobacterial infections [8, 28]. However, BCG vaccination of these individuals can confer resistance, which indicates that IL-12 signaling, and IFN-*γ* responses dependent on IL-12, may not be completely required for secondary immunity [8, 28]. Additionally, mutations in the leucine zipper domain of NF-*κ*B essential modulator (*NEMO*, also known as inhibitor of NF-*κ*B kinase subunit gamma or* IKK-γ*), encoding an intracellular protein involved in the activation of the NF-*κ*B pathway, has been demonstrated to predispose individuals to recurrent mycobacterial infections due to a lack of IL-12 production from monocytes and DCs [90].

Immunogenetics studies have also implicated other innate pathways, especially those related to pathogen sensing or cytokine and chemokine production, in immunity to mycobacterial infection. Polymorphisms in TLR2, TLR9 [91], TLR1 [92], TLR8 [93], and the intracellular signaling molecule TIRAP [94] have all been associated with susceptibility to mycobacterial infection. The mechanisms for the association between TLR and mycobacterial infection are still unclear and studies in the murine model of TB seem to indicate redundant roles for TLRs and TLR-associated molecules such as MyD88 in the generation of adaptive immune responses to Mtb [95–100]. Aside from PRRs, individuals with mutations in the inflammasome pathway have provided insight into the regulatory role of the inflammasome during mycobacterial infection. A gain of function gene variant in caspase-1 coupled with a loss of function for inhibitory caspase recruitment domain family member 8 (CARD8) promotes inflammatory diseases such as rheumatoid arthritis, but macrophages isolated from these individuals are more efficient at restricting Mtb growth* in vitro* [101]. Polymorphisms in the* Il1* gene cluster and macrophage chemoattractant protein 1 (MCP-1) also predispose individuals to TB, presumably due to an inadequate inflammatory response against infection [102–104]. Indeed, IL-1 responses in humans seem to be linked to higher eicosanoid induction that curtails excessive inflammation promoted by type I IFNs [105]. Collectively, these data suggest that the inflammasome pathway, and IL-1 in particular, may be critical in promoting enhanced immunity against Mtb in humans. In another example, a population with low serum levels of Vitamin D3 metabolites displayed increased susceptibility to active TB [106], validating* in vitro* results from human primary cells. These examples highlight the idea that immunogenetic studies on a population level are important parallel approaches that strengthen* in vitro* derived results in elucidating genes important in immunity against Mtb.

Human immunogenetic studies provide an attractive avenue for the validation of several mechanisms of resistance against Mtb observed in animal models of TB and can complement observations from* in vitro* human cells, but the innate immune response against pathogens, especially one as complex as Mtb, is multigenic and very complex. It will be interesting, and potentially very rewarding, to examine innate immune genes in studies examining individuals who are highly exposed to Mtb but who do not progress to active TB disease. This relatively resistant population, for example, health care workers in high-burden TB settings, should provide important clues to how the innate immune response may successfully handle Mtb infection.

## 4. Perspectives and Conclusions

Innate immunity is a crucial component of the immune response against Mtb but has received relatively little attention in studies of human TB. While myeloid cells serve as niches for bacterial replication, innate antimicrobial pathways activated early and throughout infection play a role in limiting disease and serve as potent regulators of antigen-specific adaptive immunity. TB disease results when pathological responses that promote chronic inflammation and lung damage dominate over protective responses that limit disease and eliminate bacteria. There is growing evidence indicating that innate immune cells are uniquely positioned to determine that balance between protective and pathogenic immune responses in human TB. However, Mtb employs myriad potent mechanisms for evading antimicrobial responses and subverting the innate immune crosstalk with adaptive immunity, thereby tilting the balance towards pathological rather than protective immune responses. Further study of human innate immune pathways during Mtb infection will be important for developing host-directed immunomodulatory therapies for TB. Further, a greater understanding of how innate immune responses impact adaptive immunity is critical for designing efficacious TB vaccines.

## References

[1] World Health Organization (2014). *Global Tuberculosis Report 2014*.

[2] Dye C., Scheele S., Dolin P., Pathania V., Raviglione M. C. (1999). Global burden of tuberculosis: estimated incidence, prevalence, and mortality by country. WHO Global Surveillance and Monitoring Project. *Journal of the American Medical Association*.

[3] Ronacher K., Joosten S. A., van Crevel R., Dockrell H. M., Walzl G., Ottenhoff T. H. M. (2015). Acquired immunodeficiencies and tuberculosis: focus on HIV/AIDS and diabetes mellitus. *Immunological Reviews*.

[4] Russell D. G. (2006). Who puts the tubercle in tuberculosis?. *Nature Reviews Microbiology*.

[5] Saunders B. M., Cooper A. M. (2000). Restraining mycobacteria: role of granulomas in mycobacterial infections. *Immunology and Cell Biology*.

[6] Russell D. G., Cardona P. J., Kim M. J., Allain S., Altare F. (2009). Foamy macrophages and the progression of the human tuberculosis granuloma. *Nature Immunology*.

[7] Orme I. M., Basaraba R. J. (2014). The formation of the granuloma in tuberculosis infection. *Seminars in Immunology*.

[8] Altare F., Durandy A., Lammas D. (1998). Impairment of mycobacterial immunity in human interleukin-12 receptor deficiency. *Science*.

[9] Fieschi C., Dupuis S., Catherinot E. (2003). Low penetrance, broad resistance, and favorable outcome of interleukin 12 receptor beta1 deficiency: medical and immunological implications. *The Journal of Experimental Medicine*.

[10] Philips J. A., Ernst J. D. (2011). Tuberculosis pathogenesis and immunity. *Annual Review of Pathology: Mechanisms of Disease*.

[11] Nouailles G., Dorhoi A., Koch M. (2014). CXCL5-secreting pulmonary epithelial cells drive destructive neutrophilic inflammation in tuberculosis. *Journal of Clinical Investigation*.

[12] Harriff M. J., Cansler M. E., Toren K. G. (2014). Human lung epithelial cells contain *Mycobacterium tuberculosis* in a late endosomal vacuole and are efficiently recognized by CD8^+^ T cells. *PLoS ONE*.

[13] Ogden C. A., deCathelineau A., Hoffmann P. R. (2001). C1q and mannose binding lectin engagement of cell surface calreticulin and CD91 initiates macropinocytosis and uptake of apoptotic cells. *The Journal of Experimental Medicine*.

[14] Rajaram M. V., Brooks M. N., Morris J. D., Torrelles J. B., Azad A. K., Schlesinger L. S. (2010). *Mycobacterium tuberculosis* activates human macrophage peroxisome proliferator-activated receptor gamma linking mannose receptor recognition to regulation of immune responses. *The Journal of Immunology*.

[15] Liu L., Liu J., Niu G., Xu Q., Chen Q. (2015). Mycobacterium tuberculosis 19-kDa lipoprotein induces Toll-like receptor 2-dependent peroxisome proliferator-activated receptor *γ* expression and promotes inflammatory responses in human macrophages. *Molecular Medicine Reports*.

[16] Mariani F., Cappelli G., Riccardi G., Colizzi V. (2000). *Mycobacterium tuberculosis* H37Rv comparative gene-expression analysis in synthetic medium and human macrophage. *Gene*.

[17] Cappelli G., Volpe P., Sanduzzi A., Sacchi A., Colizzi V., Mariani F. (2001). Human macrophage gamma interferon decreases gene expression but not replication of *Mycobacterium tuberculosis*: analysis of the host-pathogen reciprocal influence on transcription in a comparison of strains H37Rv and CMT97. *Infection and Immunity*.

[18] Nau G. J., Richmond J. F., Schlesinger A., Jennings E. G., Lander E. S., Young R. A. (2002). Human macrophage activation programs induced by bacterial pathogens. *Proceedings of the National Academy of Sciences of the United States of America*.

[19] Wang J. P., Rought S. E., Corbeil J., Guiney D. G. (2003). Gene expression profiling detects patterns of human macrophage responses following *Mycobacterium tuberculosis* infection. *FEMS Immunology and Medical Microbiology*.

[20] Volpe E., Cappelli G., Grassi M. (2006). Gene expression profiling of human macrophages at late time of infection with *Mycobacterium tuberculosis*. *Immunology*.

[21] Ragno S., Romano M., Howell S., Pappin D. J. C., Jenner P. J., Colston M. J. (2001). Changes in gene expression in macrophages infected with *Mycobacterium tuberculosis*: a combined transcriptomic and proteomic approach. *Immunology*.

[22] McGarvey J. A., Wagner D., Bermudez L. E. (2004). Differential gene expression in mononuclear phagocytes infected with pathogenic and non-pathogenic mycobacteria. *Clinical and Experimental Immunology*.

[23] Thuong N. T. T., Dunstan S. J., Chau T. T. H. (2008). Identification of tuberculosis susceptibility genes with human macrophage gene expression profiles. *PLoS Pathogens*.

[24] Mwandumba H. C., Russell D. G., Nyirenda M. H. (2004). *Mycobacterium tuberculosis* resides in nonacidified vacuoles in endocytically competent alveolar macrophages from patients with tuberculosis and HIV infection. *The Journal of Immunology*.

[25] Mwandumba H. C., Squire S. B., White S. A. (2007). Alveolar macrophages from HIV-infected patients with pulmonary tuberculosis retain the capacity to respond to stimulation by lipopolysaccharide. *Microbes and Infection*.

[26] Russell D. G., Vanderven B. C., Glennie S., Mwandumba H., Heyderman R. S. (2009). The macrophage marches on its phagosome: dynamic assays of phagosome function. *Nature Reviews Immunology*.

[27] Podinovskaia M., VanderVen B. C., Yates R. M., Coligan J. E. (2013). Dynamic quantitative assays of phagosomal function. *Current Protocols in Immunology*.

[28] Altare F., Lammas D., Revy P. (1998). Inherited interleukin 12 deficiency in a child with bacille Calmette- Guerin and *Salmonella enteritidis* disseminated infection. *The Journal of Clinical Investigation*.

[29] Picard C., Fieschi C., Altare F. (2002). Inherited interleukin-12 deficiency: IL12B genotype and clinical phenotype of 13 patients from six kindreds. *American Journal of Human Genetics*.

[30] Fabri M., Stenger S., Shin D. M. (2011). Vitamin D is required for IFN-gamma-mediated antimicrobial activity of human macrophages. *Science Translational Medicine*.

[31] Martineau A. R., Wilkinson K. A., Newton S. M. (2007). IFN-*γ*- and TNF-independent vitamin D-inducible human suppression of mycobacteria: the role of cathelicidin LL-37. *Journal of Immunology*.

[32] Liu P. T., Stenger S., Tang D. H., Modlin R. L. (2007). Cutting edge: vitamin D-mediated human antimicrobial activity against *Mycobacterium tuberculosis* is dependent on the induction of cathelicidin. *Journal of Immunology*.

[33] Liu P. T., Stenger S., Li H. (2006). Toll-like receptor triggering of a vitamin D-mediated human antimicrobial response. *Science*.

[34] Sly L. M., Lopez M., Nauseef W. M., Reiner N. E. (2001). 1Alpha,25-dihydroxyvitamin D3-induced monocyte antimycobacterial activity is regulated by phosphatidylinositol 3-kinase and mediated by the NADPH-dependent phagocyte oxidase. *The Journal of Biological Chemistry*.

[35] Rockett K. A., Brookes R., Udalova I., Vidal V., Hill A. V. S., Kwiatkowski D. (1998). 1,25-Dihydroxyvitamin D_3_ induces nitric oxide synthase and suppresses growth of *Mycobacterium tuberculosis* in a human macrophage-like cell line. *Infection and Immunity*.

[36] Crowle A. J., Ross E. J., May M. H. (1987). Inhibition by 1,25(OH)_2_-vitamin D3 of the multiplication of virulent tubercle bacilli in cultured human macrophages. *Infection and Immunity*.

[37] Rook G. A., Steele J., Fraher L. (1986). Vitamin D3, gamma interferon, and control of proliferation of *Mycobacterium tuberculosis* by human monocytes. *Immunology*.

[38] Brightbill H. D., Libraty D. H., Krutzik S. R. (1999). Host defense mechanisms triggered by microbial lipoproteins through toll-like receptors. *Science*.

[39] Jung J.-Y., Madan-Lala R., Georgieva M. (2013). The intracellular environment of human macrophages that produce nitric oxide promotes growth of mycobacteria. *Infection and Immunity*.

[40] Nicholson S., Bonecini-Almeida M. D. G., Lapa E Silva J. R. (1996). Inducible nitric oxide synthase in pulmonary alveolar macrophages from patients with tuberculosis. *The Journal of Experimental Medicine*.

[41] Thoma-Uszynski S., Stenger S., Takeuchi O. (2001). Induction of direct antimicrobial activity through mammalian toll-like receptors. *Science*.

[42] Cooper A. M., Dalton D. K., Stewart T. A., Griffin J. P., Russell D. G., Orme I. M. (1993). Disseminated tuberculosis in interferon *γ* gene-disrupted mice. *The Journal of Experimental Medicine*.

[43] Flynn J. L., Chan J., Triebold K. J., Dalton D. K., Stewart T. A., Bloom B. R. (1993). An essential role for interferon gamma in resistance to *Mycobacterium tuberculosis* infection. *The Journal of Experimental Medicine*.

[44] Ottenhoff T. H. M., Kumararatne D., Casanova J.-L. (1998). Novel human immunodeficiencies reveal the essential role of type-I cytokines in immunity to intracellular bacteria. *Immunology Today*.

[45] Chackerian A. A., Perera T. V., Behar S. M. (2001). Gamma interferon-producing CD4^+^ T lymphocytes in the lung correlate with resistance to infection with *Mycobacterium tuberculosis*. *Infection and Immunity*.

[46] Jouanguy E., Altare F., Lamhamedi S. (1996). Interferon-*γ*-receptor deficiency in an infant with fatal bacille Calmette-Guérin infection. *The New England Journal of Medicine*.

[47] Barnes P. F., Fong S.-J., Brennan P. J., Twomey P. E., Mazumder A., Modlin R. L. (1990). Local production of tumor necrosis factor and IFN-gamma in tuberculous pleuritis. *The Journal of Immunology*.

[48] Robinson D. S., Ying S., Taylor I. K. (1994). Evidence for a Th1-like bronchoalveolar T-cell subset and predominance of interferon-gamma gene activation in pulmonary tuberculosis. *American Journal of Respiratory and Critical Care Medicine*.

[49] Jouanguy E., Lamhamedi-Cherradi S., Lammas D. (1999). A human IFNGR1 small deletion hotspot associated with dominant susceptibility to mycobacterial infection. *Nature Genetics*.

[50] Newport M. J., Huxley C. M., Huston S. (1996). A mutation in the interferon-*γ*-receptor gene and susceptibility to mycobacterial infection. *The New England Journal of Medicine*.

[51] Rook G. A. W., Steele J., Ainsworth M., Champion B. R. (1986). Activation of macrophages to inhibit proliferation of *Mycobacterium tuberculosis*: comparison of the effects of recombinant gamma-interferon on human monocytes and murine peritoneal macrophages. *Immunology*.

[52] Kincaid E. Z., Ernst J. D. (2003). *Mycobacterium tuberculosis* exerts gene-selective inhibition of transcriptional responses to IFN-*γ* without inhibiting STAT1 function. *The Journal of Immunology*.

[53] Ting L.-M., Kim A. C., Cattamanchi A., Ernst J. D. (1999). Mycobacterium tuberculosis inhibits IFN-gamma transcriptional responses without inhibiting activation of STAT1. *Journal of Immunology*.

[54] Fenton M. J., Vermeulen M. W., Kim S., Burdick M., Strieter R. M., Kornfeld H. (1997). Induction of gamma interferon production in human alveolar macrophages by *Mycobacterium tuberculosis*. *Infection and Immunity*.

[55] Wang Y., Curry H. M., Zwilling B. S., Lafuse W. P. (2005). Mycobacteria inhibition of IFN-*γ* induced HLA-DR gene expression by up-regulating histone deacetylation at the promoter region in human THP-1 monocytic cells. *The Journal of Immunology*.

[56] Egen J. G., Rothfuchs A. G., Feng C. G., Horwitz M. A., Sher A., Germain R. N. (2011). ntravital imaging reveals limited antigen presentation and T cell effector function in *Mycobacterial granulomas*. *Immunity*.

[57] Eum S. Y., Kong J. H., Hong M. S. (2010). Neutrophils are the predominant infected phagocytic cells in the airways of patients with active pulmonary TB. *Chest*.

[58] Martineau A. R., Newton S. M., Wilkinson K. A. (2007). Neutrophil-mediated innate immune resistance to mycobacteria. *The Journal of Clinical Investigation*.

[59] Tan B. H., Meinken C., Bastian M. (2006). Macrophages acquire neutrophil granules for antimicrobial activity against intracellular pathogens. *Journal of Immunology*.

[60] Grakoui A., John Wherry E., Hanson H. L., Walker C., Ahmed R. (2006). Turning on the off switch: regulation of anti-viral T cell responses in the liver by the PD-1/PD-L1 pathway. *Journal of Hepatology*.

[61] Freeman G. J., Wherry E. J., Ahmed R., Sharpe A. H. (2006). Reinvigorating exhausted HIV-specific T cells via PD-1-PD-1 ligand blockade. *The Journal of Experimental Medicine*.

[62] Sharpe A. H., Wherry E. J., Ahmed R., Freeman G. J. (2007). The function of programmed cell death 1 and its ligands in regulating autoimmunity and infection. *Nature Immunology*.

[63] McNab F. W., Berry M. P., Graham C. M. (2011). Programmed death ligand 1 is over-expressed by neutrophils in the blood of patients with active tuberculosis. *European Journal of Immunology*.

[64] Berry M. P. R., Graham C. M., McNab F. W. (2010). An interferon-inducible neutrophil-driven blood transcriptional signature in human tuberculosis. *Nature*.

[65] Thome J. J. C., Yudanin N., Ohmura Y. (2014). Spatial map of human T cell compartmentalization and maintenance over decades of life. *Cell*.

[66] Wolf A. J., Linas B., Trevejo-Nuñez G. J. (2007). *Mycobacterium tuberculosis* infects dendritic cells with high frequency and impairs their function in vivo. *The Journal of Immunology*.

[67] Wolf A. J., Desvignes L., Linas B. (2008). Initiation of the adaptive immune response to *Mycobacterium tuberculosis* depends on antigen production in the local lymph node, not the lungs. *The Journal of Experimental Medicine*.

[68] Tailleux L., Neyrolles O., Honoré-Bouakline S. (2003). Constrained intracellular survival of *Mycobacterium tuberculosis* in human dendritic cells. *Journal of Immunology*.

[69] Tallieux L., Schwartz O., Herrmann J.-L. (2003). DC-SIGN is the major *Mycobacterium tuberculosis* receptor on human dendritic cells. *The Journal of Experimental Medicine*.

[70] Geijtenbeek T., Van Vliet S. J., Koppel E. A. (2003). Mycobacteria target DC-SIGN to suppress dendritic cell function. *The Journal of Experimental Medicine*.

[71] Nigou J., Zelle-Rieser C., Gilleron M., Thurnher M., Puzo G. (2001). Mannosylated lipoarabinomannans inhibit IL-12 production by human dendritic cells: evidence for a negative signal delivered through the mannose receptor. *Journal of Immunology*.

[72] Hanekom W. A., Mendillo M., Manca C. (2003). *Mycobacterium tuberculosis* inhibits maturation of human monocyte-derived dendritic cells in vitro. *Journal of Infectious Diseases*.

[73] Cella M., Engering A., Pinet V., Pieters J., Lanzavecchia A. (1997). Inflammatory stimuli induce accumulation of MHC class II complexes on dendritic cells. *Nature*.

[74] De Gassart A., Camosseto V., Thibodeau J. (2008). MHC class II stabilization at the surface of human dendritic cells is the result of maturation-dependent MARCH I down-regulation. *Proceedings of the National Academy of Sciences of the United States of America*.

[75] Hava D. L., Van Der Wel N., Cohen N. (2008). Evasion of peptide, but not lipid antigen presentation, through pathogen-induced dendritic cell maturation. *Proceedings of the National Academy of Sciences of the United States of America*.

[76] Lin M. Y., Geluk A., Smith S. G. (2007). Lack of immune responses to *Mycobacterium tuberculosis* DosR regulon proteins following *Mycobacterium bovis* BCG vaccination. *Infection and Immunity*.

[77] Aagaard C., Hoang T., Dietrich J. (2011). A multistage tuberculosis vaccine that confers efficient protection before and after exposure. *Nature Medicine*.

[78] Esin S., Counoupas C., Aulicino A. (2013). Interaction of mycobacterium tuberculosis cell wall components with the human natural killer cell receptors NKp44 and toll-like receptor 2. *Scandinavian Journal of Immunology*.

[79] Vankayalapati R., Wizel B., Weis S. E. (2002). The NKp46 receptor contributes to NK cell lysis of mononuclear phagocytes infected with an intracellular bacterium. *The Journal of Immunology*.

[80] Vankayalapati R., Garg A., Porgador A. (2005). Role of NK cell-activating receptors and their ligands in the lysis of mononuclear phagocytes infected with an intracellular bacterium. *Journal of Immunology*.

[81] Dhiman R., Indramohan M., Barnes P. F. (2009). IL-22 produced by human NK cells inhibits growth of *Mycobacterium tuberculosis* by enhancing phagolysosomal fusion. *Journal of Immunology*.

[82] Vankayalapati R., Klucar P., Wizel B. (2004). NK cells regulate CD8^+^ T cell effector function in response to an intracellular pathogen. *Journal of Immunology*.

[83] Bozzano F., Costa P., Passalacqua G. (2009). Functionally relevant decreases in activatory receptor expression on NK cells are associated with pulmonary tuberculosis in vivo and persist after successful treatment. *International Immunology*.

[84] Nirmala R., Narayanan P. R., Mathew R., Maran M., Deivanayagam C. N. (2001). Reduced NK activity in pulmonary tuberculosis patients with/without HIV infection: identifying the defective stage and studying the effect of interleukins on NK activity. *Tuberculosis*.

[85] Schierloh P., Yokobori N., Geffner L. (2009). NK cells from tuberculous pleurisy express high ICAM-1 levels and exert stimulatory effect on local T cells. *European Journal of Immunology*.

[86] Pokkali S., Das S. D., Selvaraj A. (2009). Differential upregulation of chemokine receptors on CD56^+^ NK cells and their transmigration to the site of infection in tuberculous pleurisy. *FEMS Immunology and Medical Microbiology*.

[87] Zhang R., Zheng X., Li B., Wei H., Tian Z. (2006). Human NK cells positively regulate *γδ* T cells in response to *Mycobacterium tuberculosis*. *The Journal of Immunology*.

[88] Roy S., Barnes P. F., Garg A., Wu S., Cosman D., Vankayalapati R. (2008). NK cells lyse T regulatory cells that expand in response to an intracellular pathogen. *Journal of Immunology*.

[89] Schierloh P., Aleman M., Yokobori N. (2005). NK cell activity in tuberculosis is associated with impaired CD11a and ICAM-1 expression: a regulatory role of monocytes in NK activation. *Immunology*.

[90] Filipe-Santos O., Bustamante J., Haverkamp M. H. (2006). X-linked susceptibility to mycobacteria is caused by mutations in NEMO impairing CD40-dependent IL-12 production. *The Journal of Experimental Medicine*.

[91] Velez D. R., Wejse C., Stryjewski. M. E. (2010). Variants in toll-like receptors 2 and 9 influence susceptibility to pulmonary tuberculosis in Caucasians, African-Americans, and West Africans. *Human Genetics*.

[92] Ma X., Liu Y., Gowen B. B. (2007). Full-exon resequencing reveals toll-like receptor variants contribute to human susceptibility to tuberculosis disease. *PLoS ONE*.

[93] Davila S., Hibberd M. L., Dass R. H. (2008). Genetic association and expression studies indicate a role of Toll-like receptor 8 in pulmonary tuberculosis. *PLoS Genetics*.

[94] Khor C. C., Chapman S. J., Vannberg F. O. (2007). A Mal functional variant is associated with protection against invasive pneumococcal disease, bacteremia, malaria and tuberculosis. *Nature Genetics*.

[95] Reiling N., Hölscher C., Fehrenbach A. (2002). Cutting edge: Toll-like receptor (TLR)2- and TLR4-mediated pathogen recognition in resistance to airborne infection with Mycobacterium tuberculosis. *The Journal of Immunology*.

[96] Sugawara I., Yamada H., Mizuno S., Takeda K., Akira S. (2003). Mycobacterial infection in MyD88-deficient mice. *Microbiology and Immunology*.

[97] Sugawara I., Yamada H., Li C., Mizuno S., Takeuchi O., Akira S. (2003). Mycobacterial infection in TLR2 and TLR6 knockout mice. *Microbiology and Immunology*.

[98] Scanga C. A., Bafica A., Feng C. G., Cheever A. W., Hieny S., Sher A. (2004). MyD88-deficient mice display a profound loss in resistance to *Mycobacterium tuberculosis* associated with partially impaired Th1 cytokine and nitric oxide synthase 2 expression. *Infection and Immunity*.

[99] Ryffel B., Fremond C., Jacobs M. (2005). Innate immunity to mycobacterial infection in mice: critical role for toll-like receptors. *Tuberculosis*.

[100] McBride A., Bhatt K., Salgame P. (2011). Development of a secondary immune response to *Mycobacterium tuberculosis* is independent of Toll-like receptor 2. *Infection and Immunity*.

[101] Eklund D., Welin A., Andersson H. (2014). Human gene variants linked to enhanced nlrp3 activity limit intramacrophage growth of *Mycobacterium tuberculosis*. *Journal of Infectious Diseases*.

[102] Bellamy R., Ruwende C., Corrah T., McAdam K. P. W. J., Whittle H. C., Hill A. V. S. (1998). Assessment of the interleukin 1 gene cluster and other candidate gene polymorphisms in host susceptibility to tuberculosis. *Tubercle and Lung Disease*.

[103] Wilkinson R. J., Patel P., Llewelyn M. (1999). Influence of polymorphism in the genes for the interleukin (IL)-1 receptor antagonist and IL-1*β* on tuberculosis. *The Journal of Experimental Medicine*.

[104] Flores-Villanueva P. O., Ruiz-Morales J. A., Song C. H. (2005). A functional promoter polymorphism in monocyte chemoattractant protein-1 is associated with increased susceptibility to pulmonary tuberculosis. *The Journal of Experimental Medicine*.

[105] Mayer-Barber K. D., Andrade B. B., Oland S. D. (2014). Host-directed therapy of tuberculosis based on interleukin-1 and type i interferon crosstalk. *Nature*.

[106] Wilkinson R. J., Llewelyn M., Toossi Z. (2000). Influence of vitamin D deficiency and vitamin D receptor polymorphisms on tuberculosis among Gujarati Asians in west London: a case-control study. *The Lancet*.

